# Traditional Chinese medicine combination therapy for patients with steroid-dependent ulcerative colitis: study protocol for a randomized controlled trial

**DOI:** 10.1186/s13063-016-1763-9

**Published:** 2017-01-10

**Authors:** Kai Zheng, Hong Shen, Jia Jia, Yuelin Lu, Lei Zhu, Lu Zhang, Zhaofeng Shen

**Affiliations:** 1 First Clinical Medical College, Nanjing University of Chinese Medicine, Nanjing, Jiangsu China; 2 Department of Gastroenterology, Affiliated Hospital of Nanjing University of Chinese Medicine, Nanjing, Jiangsu 210029 China; 3 Department of Endocrinology, Affiliated Hospital of Nanjing University of Chinese Medicine, Nanjing, Jiangsu China; 4 Division of Scientific Research, Affiliated Hospital of Nanjing University of Chinese Medicine, Nanjing, Jiangsu China

**Keywords:** 5-aminosalicylic acid, Azathioprine, Chinese herbal medicine, Steroid-dependent ulcerative colitis, Study protocol

## Abstract

**Background:**

Approximately 20% of patients with ulcerative colitis become steroid dependent. Azathioprine is recommended in steroid-dependent ulcerative colitis, but its side effects limit its use. Chinese herbal medicine has been widely used to treat ulcerative colitis in China. However, its effectiveness in steroid-dependent patients has not been evaluated. This study aims to investigate the efficacy of traditional Chinese medicine combination therapy with 5-aminosalicylic acid in patients with steroid-dependent ulcerative colitis.

**Methods/Design:**

This is a parallel, multicenter, randomized controlled trial. One hundred and twenty eligible patients will be randomly assigned to a traditional Chinese medicine group or azathioprine group. All patients will be given basic treatment, which includes steroids and 5-aminosalicylic acid. Patients allocated to the traditional Chinese medicine group will receive basic treatment plus Chinese herbal medicine granules, while patients in the azathioprine group will receive basic treatment plus azathioprine. The whole study will last 24 weeks. The primary outcome measure is the steroid-free remission rate. Secondary outcome measures are health-related quality of life, efficacy of endoscopic response, degree of mucosal healing, and inflammation indicators.

**Discussion:**

Results from this study may provide evidence for the effectiveness of traditional Chinese medicine combined with 5-aminosalicylic acid in patients with steroid-dependent ulcerative colitis. The findings will provide a basis for further confirmatory studies.

**Trial registration:**

Chinese Clinical Trial Register, ChiCTR-IPR-15005760. Registered on 2 January 2015.

**Electronic supplementary material:**

The online version of this article (doi:10.1186/s13063-016-1763-9) contains supplementary material, which is available to authorized users.

## Background

Ulcerative colitis is a form of inflammatory bowel disease characterized by a chronic relapsing clinical course. Intermittent rectal bleeding, diarrhea, and abdominal pain are often symptoms of ulcerative colitis. 5-Aminosalicylic acid is usually used in mild cases of ulcerative colitis. Steroids are another effective therapy for inducing remission in patients with moderate and severely active ulcerative colitis. Approximately 20–34% of patients with ulcerative colitis have chronic active disease requiring several courses of steroids to achieve remission [1, 2]. Many patients initially benefit from steroid treatment but relapse shortly after treatment cessation or after dose reduction. Up to 20% of patients with ulcerative colitis become steroid dependent [3]. The European Crohn’s and Colitis Organisation guidelines define “steroid-dependent” patients as those who are unable to reduce steroids below the equivalent of prednisolone 10 mg/day within 3 months of starting steroid use, without recurrent active disease, or who have a relapse within 3 months of stopping steroid use [4]. The risks of long-term steroid therapy include osteoporosis, pathological fractures, cataracts, metabolic changes, psychological disturbances, and infection. Moreover, steroids have not been shown to be effective for the maintenance of remission.

The American College of Gastroenterology and European Crohn’s and Colitis Organisation guidelines recommend that patients with steroid-dependent disease should be treated with azathioprine or 6-mercaptopurine [5, 6]. Azathioprine is significantly more effective than 5-aminosalicylic acid at achieving clinical and endoscopic remission in the treatment of steroid-dependent ulcerative colitis. In a single-center retrospective cohort study, 64 Asian patients with steroid-dependent inflammatory bowel disease were assessed, and 61% of patients were able to maintain clinical remission after 5 years of azathioprine therapy [7]. Toxicity induced by azathioprine and 6-mercaptopurine in patients with inflammatory bowel disease includes bone marrow suppression, drug-induced hepatitis, pancreatitis, and infection.

Complementary and alternative medicine use is common in patients with inflammatory bowel disease. The single most commonly used modality in most surveys is traditional Chinese medicine (TCM) [8]. Chinese herbal medicine is useful in improving symptoms, such as abdominal pain and diarrhea, and reducing inflammation in patients with ulcerative colitis. Many steroid-dependent patients are dissatisfied with conventional therapies, including azathioprine’s side effects; thus, they turn to TCM. However, it is not clear whether Chinese herbal medicine is effective for patients with steroid-dependent ulcerative colitis. Therefore, the primary aim of our study is to evaluate the clinical efficacy of TCM treatment among patients with steroid-dependent ulcerative colitis. Health-related quality of life in steroid-dependent patients will be a secondary aim.

## Methods/Design

**Fig. 1:**
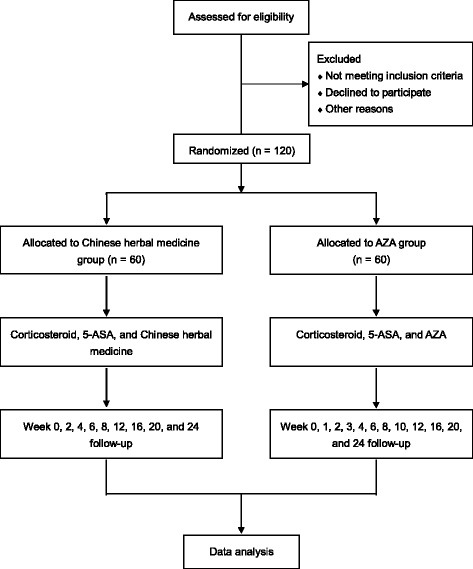
Flow chart of the trial. 5-ASA, 5-aminosalicylic acid; AZA, azathioprine

This is a multicenter, randomized controlled trial with two parallel groups and a 24-week follow-up. The centers include the Affiliated Hospital of Nanjing University of Chinese Medicine, Beijing Hospital of Chinese Medicine, Guangdong Province Hospital of Chinese Medicine, Longhua Hospital affiliated to Shanghai University of Chinese Medicine, the First Affiliated Hospital of Henan University of Chinese Medicine, Nanjing Drum Tower Hospital of Nanjing University of Medical School, Shengjing Hospital of China Medical University, the Hospital of Shanxi University of Chinese Medicine, the First Affiliated Hospital of Heilongjiang University of Chinese Medicine, and Nantong Hospital of Chinese Medicine. The study began in January 2015 and will last until October 2017. Trained researchers enroll participants. One hundred and twenty patients who meet the eligibility criteria will be recruited and allocated into two groups, with 60 individuals per group. This study has been approved by the Institutional Review Board of Human Research of the Affiliated Hospital of Nanjing University of Chinese Medicine (approval number: 2014NL-073-03). Participants must provide written informed consent to participate in the study. All participants will continue their initial steroid and 5-aminosalicylic therapy, and will be randomly assigned to receive either Chinese herbal medicine or azathioprine. The overall flow of the trial is shown in Fig. 1.

### Eligibility criteria

Participants will be considered for enrollment if they: range in age from 18 to 65 years; have a confirmed diagnosis of steroid-dependent ulcerative colitis; agree to participate in this clinical trial; and sign the informed consent form.

Patients will be excluded from the study if they: experience complications such as intestinal obstruction, intestinal perforation, toxic megacolon, or colorectal cancer; are pregnant, breast-feeding, or preparing for pregnancy; have an alanine aminotransferase level above the twice upper limit of normal; have a serum creatinine level above the upper limit of normal; have a platelet value of less than 50 × 10^9^/l; have a leukocyte count of less than 3.0 × 10^9^/l; have a known history of alcohol or drug abuse; or are enrolled in other clinical trials.

### Sample size

The remission rate for ulcerative colitis by azathioprine ranges from 59.1% to 87% [9, 10], and the average rate is about 73%. This is a noninferiority trial. For a two-sided significance level of 0.05 and power of 80% (*α* = 0.05, *β* = 0.2, *δ* = 0.15), the sample size is calculated using the formula:

$$ n=12.365\times P\left(1-P\right)/{\delta}^2 $$

Considering a 10% loss to follow-up, the sample size is 120 cases (*n* = 60 in each group).

### Randomization

A randomization sequence was generated using SAS 9.4 (SAS Institute Inc., Cary, NC, USA). An independent person (Dr. Jiandong Zou) who is not involved in observation or assessment of the patients possesses the computer-generated randomization sequence. The researchers are blinded to the randomization sequence. The randomization procedure will be conducted by research assistants using an online computerized randomization system (https://sci.njpdkj.com/pdsci/). Eligible participants will be randomly assigned to either the TCM group or azathioprine group.

### Interventions

#### Basic treatment

All eligible steroid-dependent patients will continue their initial steroid and 5-aminosalicylic therapy. Withdrawal of steroids occurs along with clinical remission. If there is clinical remission, a taper dose will be initiated with 5 mg taper weekly until a 20 mg daily dose is reached. Then tapering should proceed by 2.5 mg per week [11].

#### Chinese herbal medicine granules

Patients in the TCM group will be treated with basic treatment plus granules. The granules include *Radix Astragali* (Huangqi) 20 g, *Rhizoma Atractylodis Macrocephalae* (Baizhu) 10 g, *Semen Coicis* (Yiyiren) 20 g, *Rhizoma Dioscoreae* (Shanyao) 20 g, *Rhizoma Coptidis* (Huanglian) 6 g, *Radix Scutellariae* (Huangqin) 10 g, *Radix Pulsatillae* (Baitouweng) 10 g, *Rhizoma Smilacis Glabrae* (Tufuling) 15 g, *Radix Angelicae Dahuricae* (Baizhi) 12 g, *Radix Paeoniae Alba* (Baishao) 20 g, *Pericarpium Citri Reticulatae* (Chenpi) 12 g, *Radix Saposhnikoviae* (Fangfeng) 12 g, *Radix Aucklandiae* (Muxiang) 6 g, *Radix Sanguisorbae* (Diyu) 10 g, *Radix Arnebiae* (Zicao) 10 g, *Radix et Rhizoma Rubiae* (Qiancao) 20 g, *Fructus Alpiniae Oxyphyllae* (Yizhi) 10 g, *Rhizoma Zingiberis Preparata* (Paojiang) 6 g, and *Radix et Rhizoma Glycyrrhizae* (Gancao) 6 g. All ingredients are manufactured as a Chinese herbal granule with a weight 27.5 g (Jiangyin Tianjiang Pharmaceutical Co., Ltd, Jiangyin, China). Patients will take one Chinese herbal medicine granule per day. The course of treatment is 24 weeks unless there is a loss of follow-up.

#### Azathioprine

The control group will take basic treatment plus azathioprine. Azathioprine treatment can be initiated at a dosage of 1 mg/kg per day [12, 13]. The course of treatment will be in accordance with the TCM group.

### Outcome measures

#### Primary outcome measure

The primary outcome measure of this study is the steroid-free remission rate in patients with steroid-dependent ulcerative colitis. Remission is defined as a Mayo disease activity index score of 0–2, with no individual subscore exceeding 1 [14]. The withdrawal time of steroids will also be recorded. The outcome measure will be evaluated at the end of 24 weeks. If there is no statistical significance between the steroid-free remission rate of the TCM group and the azathioprine group, then the mean withdrawal time from steroids will be compared.

#### Secondary outcome measures

The key secondary aim is to investigate the effect of Chinese herbal medicine granules on health-related quality of life in steroid-dependent patients compared with azathioprine, as measured by the inflammatory bowel disease questionnaire. Other secondary outcome measures include the efficacy of endoscopic response, which is defined as a decrease from baseline in a Mayo disease activity index endoscopy subscore by at least 1 [14]. The degree of mucosal healing is defined as a Mayo disease activity index endoscopy subscore of 0 or 1 [15]. Improvements in inflammation indicators include fecal calprotectin, C reactive protein, and erythrocyte sedimentation rate.

### Adverse events

During the study, all adverse events, including toxicity and side effects, such as drug allergies, liver damage, renal failure, bone marrow suppression, and pancreatitis, will be reported and recorded in the case report form in detail. The researcher will report serious adverse events to the Institutional Review Board of Human Research of the Affiliated Hospital of Nanjing University of Chinese Medicine.

### Data management and statistical analysis

Data recorded in printed case report forms are kept in locked cabinets. Data will be double-entered on electronic case report forms by two persons. Frequency, median, and mean ± standard deviation of the steroid-free remission rate and the mean withdrawal time of steroids will be used for descriptive statistics. Student’s *t* test and analysis of variance will be employed using continuous variables with a normal distribution. The Mann–Whitney *U* test and Kruskal–Wallis test will be used for continuous variables with abnormal distributions, such as health-related quality of life and inflammation markers. A chi-square test or Fisher’s exact test will be used for categorical variables, including the efficacy of endoscopic response and the degree of mucosal healing. Results will be analyzed as per intention-to-treat. All data will be analyzed by an independent statistician using SAS 9.4. For all analyses, *P* < 0.05 is considered statistically significant.

To summarize the present study protocol, please see Additional file 1 for an overview of enrollment, interventions and assessments, and Additional file 2 for an overview of the Standard Protocol Items: Recommendations for Interventional Trials (SPIRIT) checklist.

## Discussion

Ulcerative colitis is a chronic inflammatory bowel disease characterized by a relapsing-remitting course owing to recurrent intestinal inflammation. Steroids are often prescribed for moderate to severe ulcerative colitis patients. Although steroids are usually effective, long-term steroid use should be avoided because of its severe side effects. Patients who initially respond to steroids but then relapse after tapering or shortly after discontinuation, and require reintroduction of steroids to induce remission are defined as steroid dependent. The therapeutic objective of steroid-dependent patients is to achieve steroid-free remission.

In view of the side effects of steroids, different drugs are used in the management of steroid-dependent ulcerative colitis. Drugs used include established agents, such as thiopurines, methotrexate, infliximab, adalimumab, vedolizumab, and golimumab. If common treatments fail, colectomy may be performed [16]. Current guidelines recommend thiopurines as first-line therapy to spare steroids in steroid-dependent ulcerative colitis [3, 6]. Therefore, the thiopurine agents azathioprine and 6-mercaptopurine are used to sustain steroid withdrawal in patients with steroid-dependent ulcerative colitis [5]. Azathioprine is a purine analog that competitively inhibits the biosynthesis of purine ribonucleotides [17]. Azathioprine is the main therapy for steroid-dependent patients in China, because vedolizumab and golimumab are not available, and adalimumab is not indicated for use in patients with ulcerative colitis. Furthermore, cessation of infliximab is still considered in clinical practice because of its high cost and fear of long-term side effects [18]. There is also a reduced response to infliximab, which may reach approximately 13% per year with uninterrupted scheduled maintenance therapy [19]. Fraser *et al.* reported that the remission rate was 87% in 346 ulcerative colitis patients who received more than 6 months of azathioprine treatment [10]. Another study demonstrated that the steroid-free remission rate was 59.1% in patients with steroid-dependent or steroid-resistant ulcerative colitis treated with azathioprine [9]. The most common adverse event of azathioprine is leukopenia, which occurs in 3.8% of patients [20]. Other adverse events include hepatitis, infection, pancreatitis, and hair loss. Once absorbed into the plasma, azathioprine is nonenzymatically converted to 6-mercaptopurine. 6-Mercaptopurine can be metabolized to 6-methylmercaptopurine by the enzyme thiopurine methyltransferase (TPMT), to 6-thiouric acid by the enzyme xanthine oxidase, and to the active metabolites of 6-thioguanine nucleotide. 6-Thioguanine nucleotide is the active ribonucleotide of 6-mercaptopurine; it functions as a purine antagonist, resulting in immunosuppression and lymphocytotoxicity. A deficiency in TPMT may lead to preferential 6-mercaptopurine metabolism into 6-thioguanine nucleotide, resulting in myelosuppression [21]. An apparent genetic polymorphism of TPMT is associated with TPMT enzyme deficiency. Despite a lower frequency of variant TPMT observed in Asian populations [22], lower starting doses of azathioprine are recommended in Asian populations than in white populations, along with close monitoring of complete blood count and liver function [13].

Chinese herbal medicine is a type of complementary and alternative medicine, and is often used in patients with inflammatory bowel disease [8], especially in China. Dai *et al.* [23] analyzed 247 patients with ulcerative colitis. The Sutherland Disease Activity Index scores for patients treated with TCM and those treated with integrated TCM and Western medicine were significantly lower for both groups after treatment (*P* < 0.01). Sugimoto *et al.* used the Chinese herbal medicine Qing-Dai to treat 20 patients with moderate ulcerative colitis. The rates of clinical response, clinical remission, and mucosal healing were 72%, 33%, and 61% at week 8, respectively [24]. Qing-Dai stimulates mucosal type 3 innate lymphoid cells to generate interleukin-22, which induces antimicrobial peptide and tight junction production, suggesting a therapeutic mechanism for mucosal healing [25]. Our study using Chinese herbal medicine plus 5-aminosalicylic in patients with steroid-dependent ulcerative colitis aims to observe improvements in the primary outcome of steroid-free remission rate, and the secondary outcomes of health-related quality of life, endoscopic response, mucosal healing, and inflammation indicators. We expect that the steroid-free remission rate of the TCM group will be improved over that of the azathioprine group. The mean withdrawal time of steroids in the TCM groups should be less than that of the azathioprine group, which would reduce the risks of steroid therapy. We anticipate that the results will show that Chinese herbal medicine is effective for patients with steroid-dependent ulcerative colitis.

There are several limitations to our study. First, this is an open-label randomized controlled trial without double blinding. Because of the lack of placebo control groups, our findings will be interpreted with caution. Another limitation to this study is the lack of TPMT and 6-thioguanine nucleotide testing. Some patients do not respond to azathioprine because of lower 6-thioguanine nucleotide levels. Although a lower frequency of variant TPMT is observed in Asian populations, TPMT and 6-thioguanine nucleotide testing may assist dose optimization of azathioprine to achieve response and avoid drug-induced toxicity. Both TPMT and 6-thioguanine nucleotide testing will be determined in corollary studies. The last limitation is of first-choice therapy in steroid-dependent ulcerative colitis. The Toronto consensus in 2015 recommends anti-TNF therapy to induce and maintain complete steroid-free remission in patients with steroid-dependent ulcerative colitis [26]. The consensus group also recommends that anti-TNF plus azathioprine therapy is the preferred choice for steroid-dependent patients rather than monotherapy to induce complete remission [26, 27]. However, azathioprine is still the most practicable therapy for steroid-dependent patients in China, because anti-TNF agents are not widely used. Combination therapy with anti-TNF agents will be available in the future. Notwithstanding its limitations, this study will suggest whether Chinese herbal medicine can be an effective complementary and alternative medicine in patients with steroid-dependent ulcerative colitis.

## Trial status

The first patient in the study was enrolled on 24 April 2015. The trial is enrolling participants.

## Additional files

Additional file 1: Schedule of enrollment, interventions, and assessments. (DOC 148 kb)
Additional file 2: SPIRIT 2013 Checklist: recommended items to address in a clinical trial protocol and related documents. (DOC 133 kb)

## Abbreviations

TCM: traditional Chinese medicine
TNF: tumor necrosis factor
TPMT: thiopurine methyltransferase
