# Supplementary material for: Traditional Chinese medicine combination therapy for patients with steroid-dependent ulcerative colitis: study protocol for a randomized controlled trial
Source: Trials. 2017 Jan 10;18:8. doi: 10.1186/s13063-016-1763-9 (PMC5223474; doi:10.1186/s13063-016-1763-9)
Supplement: Additional file 1: — Schedule of enrollment, interventions, and assessments. (DOC 148 kb) [file 13063_2016_1763_MOESM1_ESM.doc]

Figure S1: Schedule of enrollment, interventions, and assessments

| **TCM Group** | **STUDY PERIOD** | | | | | | | | | |
| --- | --- | --- | --- | --- | --- | --- | --- | --- | --- | --- |
|  | **Enrolment** | **Allocation** | **Post-allocation** | | | | | | | **Close-out** |
| **TIMEPOINT** | **Week -2-0** | **Week 0** | **Week 2** | **Week 4** | **Week 6** | **Week 8** | **Week 12** | **Week 16** | **Week 20** | **Week 24** |
| **ENROLMENT:** |  |  |  |  |  |  |  |  |  |  |
| Eligibility screen | X |  |  |  |  |  |  |  |  |  |
| Informed consent | X |  |  |  |  |  |  |  |  |  |
| Allocation |  | X |  |  |  |  |  |  |  |  |
| **INTERVENTIONS:** |  |  |  |  |  |  |  |  |  |  |
| Basic treatment |  |  |  |  |  |  |  |  |  |  |
| Chinese herbal medicine |  |  |  |  |  |  |  |  |  |  |
| **ASSESSMENTS:** |  |  |  |  |  |  |  |  |  |  |
| Symptoms and signs |  | X | X | X | X | X | X | X | X | X |
| HRQOL score |  | X |  |  |  |  |  |  |  | X |
| Mayo score |  | X |  |  |  |  |  |  |  | X |
| Colonoscopy and pathology | X |  |  |  |  |  |  |  |  | X |
| ESR | X |  | X | X |  | X | X | X |  | X |
| CRP | X |  | X | X |  | X | X | X |  | X |
| Fecal calprotectin | X |  | X | X |  | X | X | X |  | X |
| CBC | X |  | X | X |  | X | X | X |  | X |
| Urinalysis | X |  |  |  |  |  |  |  |  | X |
| Stool Routine and occult blood test | X |  | X | X | X | X | X | X | X | X |
| Hepatic function | X |  | X | X |  | X | X | X |  | X |
| Renal function | X |  | X | X |  | X | X | X |  | X |
| ECG | X |  |  |  |  |  |  |  |  | X |

| **AZA Group** | **STUDY PERIOD** | | | | | | | | | | | | |
| --- | --- | --- | --- | --- | --- | --- | --- | --- | --- | --- | --- | --- | --- |
|  | **Enrolment** | **Allocation** | **Post-allocation** | | | | | | | | | | **Close-out** |
| **TIMEPOINT** | **Week -2-0** | **Week 0** | **Week 1** | **Week 2** | **Week 3** | **Week 4** | **Week 6** | **Week 8** | **Week 10** | **Week 12** | **Week 16** | **Week 20** | **Week 24** |
| **ENROLMENT:** |  |  |  |  |  |  |  |  |  |  |  |  |  |
| Eligibility screen | X |  |  |  |  |  |  |  |  |  |  |  |  |
| Informed consent | X |  |  |  |  |  |  |  |  |  |  |  |  |
| Allocation |  | X |  |  |  |  |  |  |  |  |  |  |  |
| **INTERVENTIONS:** |  |  |  |  |  |  |  |  |  |  |  |  |  |
| Basic treatment |  |  |  |  |  |  |  |  |  |  |  |  |  |
| AZA |  |  |  |  |  |  |  |  |  |  |  |  |  |
| **ASSESSMENTS:** |  |  |  |  |  |  |  |  |  |  |  |  |  |
| Symptoms and signs |  | X | X | X | X | X | X | X | X | X | X | X | X |
| HRQOL score |  | X |  |  |  |  |  |  |  |  |  |  | X |
| Mayo score |  | X |  |  |  |  |  |  |  |  |  |  | X |
| Colonoscopy and pathology | X |  |  |  |  |  |  |  |  |  |  |  | X |
| ESR | X |  |  | X |  | X |  | X |  | X | X |  | X |
| CRP | X |  |  | X |  | X |  | X |  | X | X |  | X |
| Fecal calprotectin | X |  |  | X |  | X |  | X |  | X | X |  | X |
| CBC | X |  | X | X | X | X | X | X | X | X | X | X | X |
| Urinalysis | X |  |  |  |  |  |  |  |  |  |  |  | X |
| Stool Routine and occult blood test | X |  | X | X | X | X | X | X | X | X | X | X | X |
| Hepatic function | X |  |  | X |  | X |  | X |  | X | X |  | X |
| Renal function | X |  |  | X |  | X |  | X |  | X | X |  | X |
| ECG | X |  |  |  |  |  |  |  |  |  |  |  | X |
